# High-rate transition metal-based cathode materials for battery-supercapacitor hybrid devices

**DOI:** 10.1039/d1na00523e

**Published:** 2021-07-30

**Authors:** Cong Wang, Zehao Song, Pei Shi, Lin Lv, Houzhao Wan, Li Tao, Jun Zhang, Hanbin Wang, Hao Wang

**Affiliations:** Hubei Yangtze Memory Labs, School of Microelectronics, Hubei University Wuhan 430000 PR China houzhaow@hubu.edu.cn wangh@hubu.edu.cn nanoguy@126.com

## Abstract

With the rapid development of portable electronic devices, electric vehicles and large-scale grid energy storage devices, there is a need to enhance the specific energy density and specific power density of related electrochemical devices to meet the fast-growing requirements of energy storage. Battery-supercapacitor hybrid devices (BSHDs), combining the high-energy-density feature of batteries and the high-power-density properties of supercapacitors, have attracted mass attention in terms of energy storage. However, the electrochemical performances of cathode materials for BSHDs are severely limited by poor electrical conductivity and ion transport kinetics. As the rich redox reactions induced by transition metal compounds are able to offer high specific capacity, they are an ideal choice of cathode materials. Therefore, this paper reviews the currently advanced progress of transition metal compound-based cathodes with high-rate performance in BSHDs. We discuss some efficient strategies of enhancing the rate performance of transition metal compounds, including developing intrinsic electrode materials with high conductivity and fast ion transport; modifying materials, such as inserting defects and doping; building composite structures and 3D nano-array structures; interfacial engineering and catalytic effects. Finally, some suggestions are proposed for the potential development of cathodes for BSHDs, which may provide a reference for significant progress in the future.

## Abstract 2

With the rapid development of portable electronic devices and large-scale grid energy storage devices, it is need to enhance the specific energy and power density of electrochemical devices to meet the fast-growing requirements of energy storage.

## Graphical abstract

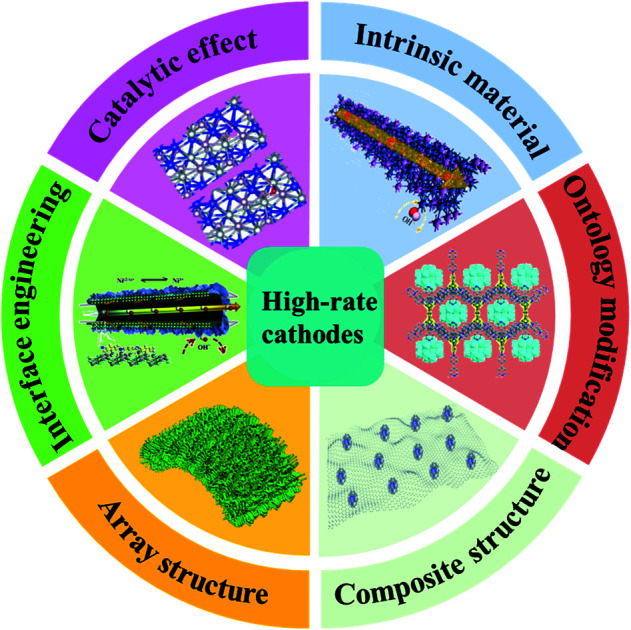

## 1. Introduction

**Fig. 1:**
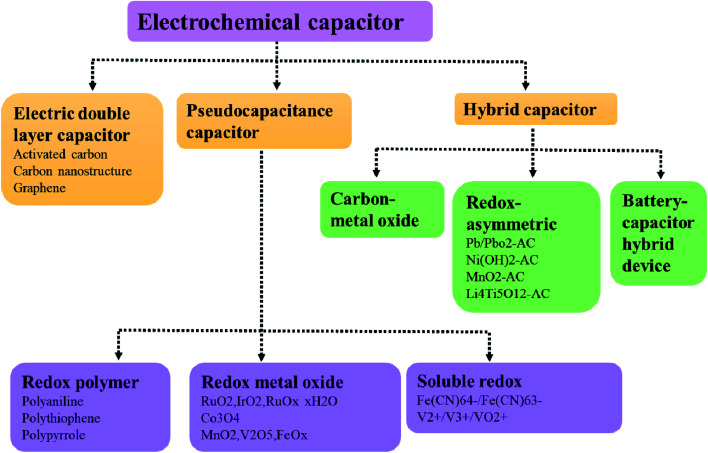
Classification of electrochemical capacitors.

**Fig. 2:**
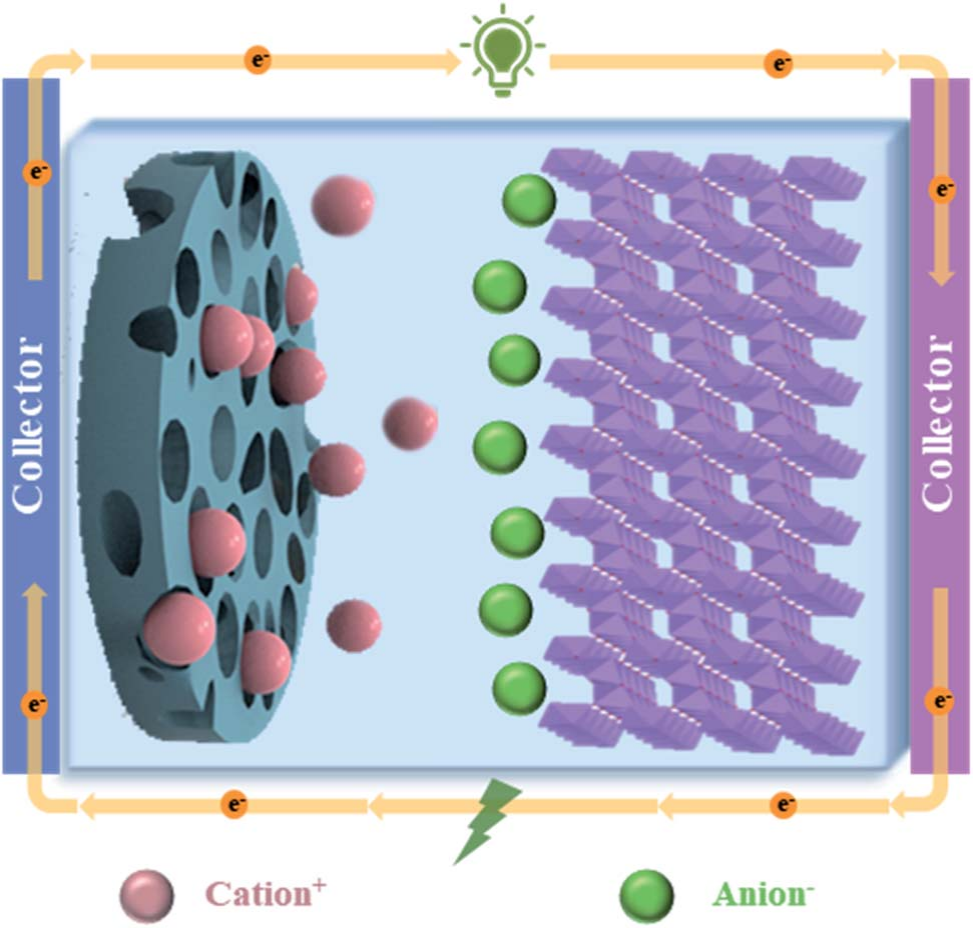
Schematic diagram of the working mechanism in BSHDs.

The fast-growing economy of modern society has caused transitional consumption of primary energy sources accompanied by alarming environmental pollution, thus making it crucial to create high-performance energy conversion/storage devices.^1,2^ Energy storage devices should have the ability of supporting the national grid, meeting the expected increment of the global energy demand, and also matching the expanding requirements of the transportation sector in terms of electrification, electric vehicles, *etc.*^3^ In the past few decades, lithium-ion batteries have been widely used as a charging power source in portable electronic devices due to their high energy density and high operating voltage. But present lithium-ion batteries are not able to meet some of the new emerging demands, such as fast charging, due to their low power density.^4–6^ Supercapacitors are an ideal energy storage device because of some favourable features, including higher power density than batteries, and higher energy density than ordinary dielectric capacitors.^7^ Based on the mechanism of energy storage, supercapacitors can be divided into three categories: double layer capacitors, Faraday quasi-capacitors and battery-super capacitor hybrid devices (BSHDs) (Fig. 1).^8^ Among them, BSHDs attract more attention because they combine the high-energy-density feature of batteries and the high-power-density properties of supercapacitors. This should be attributed to the special structure where battery-type and capacitor-type electrode materials are applied in the cathode and anode, corresponding to the energy storage mechanism of the redox reaction and double layer, respectively (Fig. 2). Obviously, battery-type cathode materials are the key to achieving high-performance BSHDs.^9,10^

Battery-type cathodes mainly rely on transition metal compounds, such as metal oxides, hydroxides, sulfides, phosphides, *etc.*, which are capable of providing high specific capacity due to their rich redox reactions.^11^ The electrochemical properties of different transition metal compounds are summarized in Table 1. Nickel oxide (NiO) has received much attention among transition metal oxides because of its excellent redox reversibility, high theoretical specific capacity (2584 F g^−1^),^12^ wide potential window and low cost.^13^ But NiO has the drawbacks of poor electrical conductivity, slow ion diffusion rates and the change of volume during charge and discharge, inducing impaired rate performance and cycling stability.^14,15^ Transition metal hydroxides are challenged by similar problems.^16^ In contrast, transition metal sulfides are a new generation of active materials for supercapacitors. For example, cobalt sulfide has excellent electrochemical properties, high theoretical capacity, structural diversity and multiple valence states and is widely applied in electrochemistry in electrocatalysts, lithium-ion batteries, and supercapacitors.^17–19^ Compared to oxygen or sulfur, phosphorus has lower electronegativity. Thus, transition metal phosphides show quasi-metallic structures and good electrical conductivity,^20^ exhibiting better electrochemical response than transition metal oxides/sulfides.^21–23^ However, due to the poor-electrical conductivity and slow ion transport kinetics of these materials, the poor-rate performance causes the trade-off of high power density and high energy density.

**Table 1:** Electrochemical properties of different types of transition metal-based compounds

Type	Electrode materials	Specific capacity (F g^−1^) at 1 A g^−1^	Specific capacity (F g^−1^) at 10 A g^−1^	Rate performance	Stability	Ref.
Oxide	NiO	473	332.8	70%	94%, *n* = 3000	24
	CoO	352	280	79.5%	92.9%, *n* = 5000	25
	Co_3_O_4_	1121	1023	91%	98.2%, *n* = 6000	26
	NiCo_2_O_4_	1516	1436	94.7%	93%, *n* = 2000	27
Hydroxide	Ni(OH)_2_	2110	1171	55.5%	53%, *n* = 2000	28
	Ni Co-LDH	986.3	537.5	54.5%	92.3%, *n* = 10 000	29
	α-Co(OH)_2_	1310	960	73.3%	83.9, *n* = 4000	30
	β-Ni(OH)_2_	1065.7	812.5	76.2%	91%, *n* = 10 000	31
Sulfide	NiS	1315.4	677.1	51.5%	89.2%, *n* = 5000	32
	CoS	335	128	38%	87.3%, *n* = 2000	33
	CoS_2_	936	746	79.7%	83%, *n* = 5000	34
	Co_9_S_8_	1852	1228	66.3%	86%, *n* = 5000	35
	NiCoS	1653	1344	81.3%	84%, *n* = 3000	36
	NiCo_2_S_4_	2161.7	1660	76.8%	92.4%, *n* = 10 000	37
Selenide	Ni_0.85_Se	3150	1460	46%	90.1, *n* = 5000	38
	Co_0.85_Se	1528	894.4	58.5%	92, *n* = 5000	39
	Ni_0.6_Co_0.4_Se_2_	1580	1320	83.5%	90, *n* = 20 000	40
	Ni_0.67_Co_0.33_Se_2_	784.2	674.4	86%	97%, *n* = 2000	41
Phosphide	Ni_2_P	404.2	304.3	75.3%	81%, *n* = 2000	42
	Ni_12_P_5_	1414.4	963.4	68%	53%, *n* = 1000	43
	CoP	558	462	82.8%	98%, *n* = 5000	44
	NiCoP	1258	1056	83.9%	90.1, *n* = 10 000	45

Rate performance is an important index that characterizes the electrochemical properties of materials and devices, depending on the reversibility of redox reactions occurring on the surface and the structural stability of electrode materials.^46,47^ Thus, the enhancement of rate performance is beneficial to improve the electrochemical performance of BSHDs and effectively promote their practical applications. In order to improve the rate performance of electrode materials, firstly with respect to kinetics and thermodynamics, they need to achieve high electrical conductivity and high surface activity. Secondly, some strategies such as doping, inserting defects, coupling with conducting materials, and building core–shell heterostructures can effectively improve the electrochemical activity of electrode materials and accelerate the kinetics of electrochemical reactions, thus leading to high-rate performance of electrode materials. For instance, Yu *et al.* reported urchin-like nickel–cobalt phosphide hollow spheres with polymetallic redox centers and excellent electrical conductivity, exhibiting a specific capacity of 761 C g^−1^ at a current density of 1 A g^−1^ and a capacity retention of 91.1% at 20 A g^−1^.^48^ Liu *et al.* reported composites achieved by integrating nickel–cobalt bimetallic hydroxide nanosheets with functionalized graphite.^49^ The fast electron/ion transport channel enabled excellent rate performance of the composite electrode, that is, specific capacities of 2442 F g^−1^ and 2039 F g^−1^ were achieved at current densities of 1 A g^−1^ and 50 A g^−1^, respectively, accompanied by a capacity retention of 83.5%. Cao *et al.* fabricated oxygen-rich defective Co_3_O_4_/graphene composites, whose oxygen vacancies on the surface can promote electrochemical charge transfer by generating another defect level in the band gap, thus significantly enhancing the rate performance of the electrode material.^50^ The corresponding specific capacity was 978.1 F g^−1^ at 1 A g^−1^, and a capacity retention of 93.7% was achieved even at 10 A g^−1^. Although many studies have been carried out based on high-rate transition metal compound-based electrodes, they usually focus on one individual material (*e.g.* transition metal oxides), or one single property (*e.g.* porous materials), or one structure (*e.g.* hierarchically structured oxides). Nevertheless, some reviews have discussed the strategies of how to enhance the stability of transition metal compounds. Since these previous reviews have mainly focused on partial discussions, there is a need for a systematic and comprehensive review.

The purpose of this review is to provide our perspective on improving the rate performance of transition metal compound-based cathodes in BSHDs, and offer some concluding remarks and suggestions for future development, which may prove to be a reference for further research work.

## 2. Energy storage mechanism of high-rate electrode materials

**Fig. 3:**
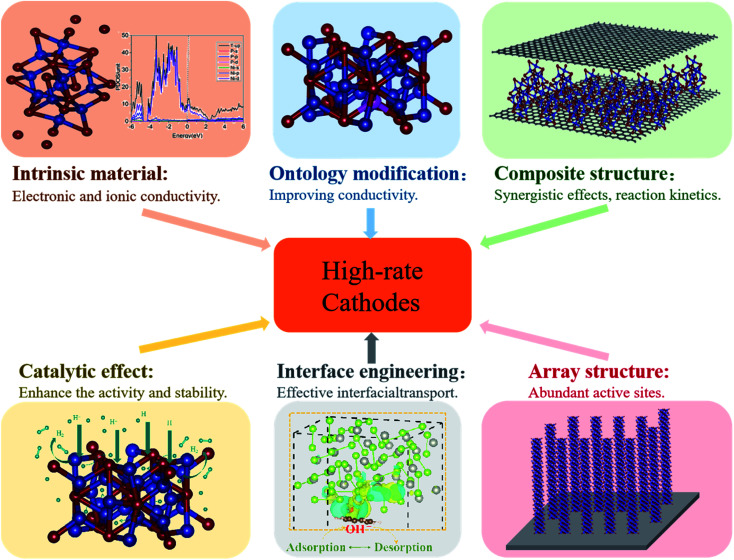
Methods of enhancing the rate performance of transition metal compounds.

Low electrical conductivity and ion diffusion kinetics of transition metal compounds induce a poor rate performance, which is unable to meet the requirement for high energy density. To overcome this barrier, some efficient methods of enhancing the rate performance have been established recently, including (1) finding intrinsic materials exhibiting fast electron/ion transport, (2) modifying intrinsic materials (*i.e.* inserting defects or doping), (3) building composite structures, (4) synthesizing three-dimensional (3D) designed nano-array structures, (5) interface engineering and (6) utilizing the catalytic effects of transition metal compounds (Fig. 3).

Fast electron/ion transport in intrinsic materials boosts charge transport and ion diffusion, enabling fast and reversible Faraday reactions at large current density, and thus enhancing electrochemical energy storage.^51,52^ In recent years, spinel-type transition metal compound AB_2_M_4_ (where M is O, S) has been found to possess higher electrical conductivity and electrochemical activity than single metal oxides/sulfides, and is therefore considered a promising material for high-rate cathodes in BSHDs.^18,53–56^ Specifically, high conductivity would enable rapid electron transfer, and the synergistic effect of A and B ions can provide a richer Faraday redox reaction during the charging and discharging process.^57–61^ Besides the enhancement of electron transfer, the achievement of fast ion transport in the entire system is also another effective approach to improve the rate performance of electrode materials. One of the corresponding methods is to synthesize porous hollow nanostructures, which are able to exhibit superior performance to their bulk structure.^48,62^ Because porous hollow nanostructures have more active sites than their bulk counterpart, a larger specific surface area would be used in charge storage;^48,63–65^ the contact area between the electrolyte and active material would be increased and electrolyte penetration would be accelerated;^66,67^ shortening the ion diffusion path, promoting the transfer efficiency of ions,^18,68,69^ reducing the internal resistance, and consequently inducing higher rate performance.^62,70,71^ However, most transition metal compounds are limited by slow electron and ion transport.

Modification of intrinsic materials can improve the quality of the interface between the electrode and electrolyte, and provides additional Faraday pseudocapacitance as well, thus enabling high capacity storage.^72–74^ The modification methods include building defects and doping. Building defects is able to effectively break the periodic structure inside the crystal and alter the charge distribution in the surrounding, further changing the physicochemical properties of the electrode material.^75–77^ Element doping will modulate the band gap and the conductivity of electrode materials, thus reinforcing the rate performance.^78–81^ In addition, modification can reconfigure the crystalline structure inside the electrode material, which would work as a buffer, avoiding the capacity loss caused by volume expansion of electrode materials during a fast charging and discharging process.^31^

Composite structures can effectively combine the advantages of different materials to meet the demand of high rate performance. For transition metal compounds, many previous research studies have demonstrated an efficient approach of improving rate performance *via* coupling carbon-based materials. Among such carbon-based materials, graphene is one of the most widely applied, owing to its excellent electrical conductivity and huge specific surface area. Such composites would take their advantages (such as the synergistic effect from components and fast electron transport) to accelerate the speed of charge transfer and ion diffusion,^19,47,82,83^ mitigating the volume expansion during the redox reaction.^56,84–86^ Therefore, they are able to greatly enhance the rate performance of electrode materials with the improvement of cycling stability.^87,88^ Another approach of optimizing energy storage performance is to build heterojunctions using different transition metal compounds (such as Co_3_O_4_–RuO_2_,^89^ and MoSe_2_–Ni(OH)_2_ (ref. 70)), which will provide a built-in electric field to facilitate the transfer of electrons by an electrostatic force, finally improving the kinetics of the redox reaction during charging and discharging, and thus achieving high rate performance.^90^

3D nano-array structures can utilize active materials as much as possible and provide rich redox reaction sites, further increasing the rate performance. As the quality of the electron transporting channel is crucial to the performance of electrode materials, the morphologies of 3D nanostructures would play an important role in achieving high-performance electrode materials.^91^ It has been demonstrated that 3D nano-array structures consisting of 1D nanorods and 2D nanosheets have huge potential in enhancing the electrochemical properties of electrode materials. 2D nanosheets exhibit an enormous surface area, which will provide abundant active sites for redox reactions, whereas 1D nanorods have a large contact area with electrolyte ions during the electrochemical reactions. Besides, 1D nanorods not only act as an electronic highway to transfer electrons from the active material to the external circuit, but also have the ability of mitigating volume expansion.

The interface is an important position where electrochemical reactions occur in energy storage devices, and the performance of BSHD electrode materials strongly relies on effective electron and ion transport on the surface. The construction of high-quality interfaces inside composite electrodes or between the electrode/electrolyte is a key step of enhancing the electrochemical energy storage performance.^92^ In addition, catalyst materials play a crucial role in redox reactions that occur during the storage and conversion of electrochemical energy. The activity and stability of catalyst materials determine the electrochemical performance of energy storage devices, more or less. Therefore, it is important to survey the catalytic characteristics of electrode materials improving the rate performance and cycling stability.

**Fig. 4:**
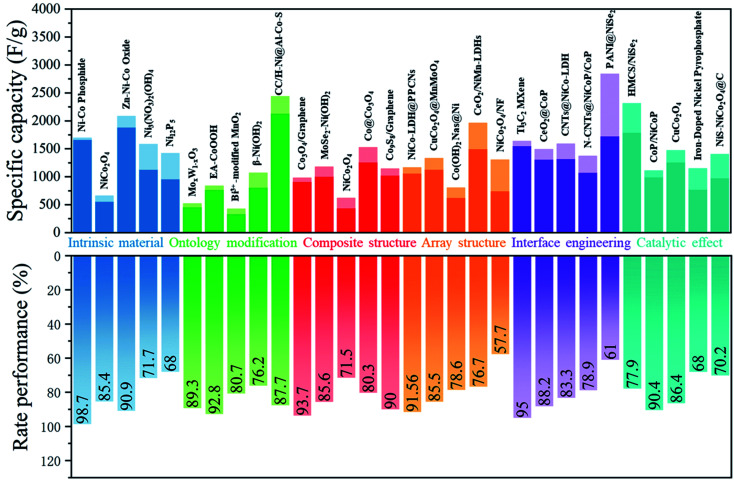
Effective methods used in increasing the rate performance of transition metal based cathodes in BSHDs.

Here, we will carry out a more detailed discussion with respect to individual strategies from the above arguments. Fig. 4 summarizes the rate performance of some transition metal compound cathodes based on different strategies.

## 3. Strategies of enhancing the rate performance of transition metal compound-based cathodes

### 3.1 Intrinsic materials

#### 3.1.1 High-conductivity electrode materials

Common battery-type transition metal compounds, such as nickel oxide,^93^ cobalt oxide,^25,94^ nickel sulfide,^95,96^ and cobalt phosphide,^97^ used as cathode materials usually have poor electrical conductivity, slow ion diffusion, and bad cycling stability, thus inducing low rate performance. Such transition metal compounds with low rate performance are not able to meet the needs and greatly limit practical applications.^98^ Therefore, researchers have dedicated themselves to find strategies of synthesizing high conductivity electrode materials, which can make electrons transfer from the collector to the surface of the electrode material faster, consequently reducing the charge transfer resistance.^99^ Among a lot of bimetallic compounds, nickel–cobalt bimetallic oxides are one of the most promising materials for high-performance pseudocapacitive electrodes. Compared to monometallic oxides, such as nickel oxide and cobalt oxide, spinel structure-based nickel–cobalt bimetallic oxides are able to offer more abundant redox active centers due to the synergistic effect, and thus higher specific capacity.^54^ Jiang *et al.* prepared NiCo_2_O_4_ and used it as an electrode material, which also showed high conductivity enabling excellent rate performance and cycling stability. It has a specific capacity of 658 F g^−1^ at a current density of 1 A g^−1^, and still kept 78% capacity retention even when the current density was increased 20 times.^61^ Lin *et al.* prepared a NiCo_2_S_4_ electrode material effectively improving the interaction between the electrolyte and active material, accelerating electron transfer during charge and discharge, and thus exhibiting impressive capacity and rate performance (*i.e.* a capacity of 82.6 mA h g^−1^ at 1 A g^−1^ and even a capacity of 46.2 mA h g^−1^ at 16 A g^−1^).^18^

**Fig. 5:**
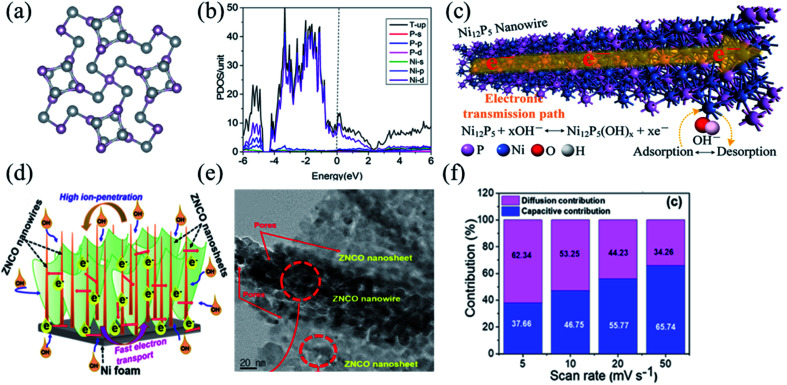
(a) The crystal structure of Ni_12_P_5_. (b) The projected density of states of the Ni_12_P_5_ bulk counterpart. (c) Electron transporting mechanism of Ni_12_P_5_ nanowires. Panel a–c adapted with permission from ref. 43, copyright 2019, Elsevier. (d) Electron transporting mechanism of ZNCO nanostructures. (e) The HRTEM image of the ZNCO. (f) Contribution of the capacitive- and diffusion-controlled process for the ZNCO electrode at different scanning rates. Panels d–f adapted with permission from ref. 112, copyright 2020, American Chemical Society.

With the electronegativity of the atom P in transition metal compounds being lower than that of the atoms O and S, transition metal phosphides have faster electron transfer and more active redox reactions enabling better electrochemical properties.^98^ Further, larger ionic gaps in the atomic structure of transition metal phosphides would enable more efficient electron/ion transport and better electrical conductivity, and thus transition metal phosphides would have good prospects as cathode materials in BSHDs. Wang *et al.* successfully synthesized high conductivity Ni_12_P_5_ nanowires by a simple one-step hydrothermal method. Such Ni_12_P_5_ nanowires can provide abundant electrochemical active centers and excellent electron transport paths.^43^ The electrode material showed a reversible specific capacity of 707.2 C g^−1^ at 1 A g^−1^ and a specific capacity of 481.7 C g^−1^ at 10 A g^−1^ with good capacity retention. Fig. 5a illustrates the crystal structure of Ni_12_P_5_, wherein the high porosity enables a high conductivity of 10 S cm^−1^. Fig. 5b demonstrates the density of states of Ni and P, suggesting that the energy bands of Ni_12_P_5_ are mainly attributed to Ni_2p_ and P_2p_. The partial overlap of electron densities on the Fermi energy level further confirms the high conductivity of Ni_12_P_5_, which is the reason for high-rate performance. Fig. 5c shows the mechanism of electron transport in Ni_12_P_5_ nanowires in which the adsorption/desorption of OH^−^ on the surface of the Ni_12_P_5_ nanowires promotes electron transfer; the corresponding redox reaction on the surface of the Ni_12_P_5_ nanowires follows eqn (1):

1 Ni_12_P_5_ + *x*OH^−^ ↔ Ni_12_P_5_(OH)_*x*_ + *x*e^−^

#### 3.1.2 Highly-active-site electrode materials

To improve the rate performance of transition metal compound-based cathodes in BSHDs, another effective strategy is to prepare nanostructures with highly active sites. One of the simple approaches is to transform the bulk into porous nano-hollow structures, which would enable superior physical and chemical properties to the bulk materials.^48,62^ Hollow nanostructures can facilitate penetration of the electrolyte into the interior of electrode materials, increasing the amount of electrochemically active sites, and providing a large specific surface area for charge storage.^60,61,100^ Further, the large specific surface area would shorten the transport paths of ions and electrons, reducing the internal resistance, and thus enhancing the rate performance of the electrode material.^91,101,102^ The pores in electrode materials can increase the contact area between the electrolyte and electrode, accelerating the kinetics of electrochemical reactions, and finally ensuring rapid and effective charge storage reactions.^103^

Among battery-type cathode materials, Co_3_O_4_ is considered a promising electrode material due to its high ideal specific capacity, good electrochemical stability and redox activity.^104,105^ However, the rapid redox reaction causes severe attenuation of specific capacity during charging and discharging at large current density. To solve this problem, Sun *et al.* synthesized ultrathin Co_3_O_4_ nanosheets with high electrochemical activity and stability based on Co-MOFs as precursors.^26^ The corresponding specific capacities were 1121 F g^−1^ at 1 A g^−1^ and 873 F g^−1^ at 25 A g^−1^ with a capacity retention of 77.9%. The Co_3_O_4_ nanosheets increase the contact area with the electrolyte, promoting the diffusion of ions in the electrode material, accelerating the reaction kinetics, and resulting in good specific capacity performance at high current densities.^106,107^ In addition, the unique structure of Co_3_O_4_ nanosheets benefits the mitigation of structural collapse caused by volume changes during redox cycling, providing better reversible capacity and cycling stability.

Compared to monometallic/bimetallic compounds, ternary metal compounds have been widely used in energy storage systems because of higher electrochemical properties from the synergistic effect of multiple metal ions.^108–111^ Kim *et al.* reported a unique 3D hierarchical nanostructure of zinc–nickel–cobalt ternary oxide (ZNCO) (Fig. 5e).^112^ The synergistic effect between nanowires and nanosheets would provide abundant active sites for redox electrochemical reactions and reduce the volume expansion and mechanical strain of electrode materials during long-term charge/discharge cycles (Fig. 5d). Fig. 5f illustrates the contribution from diffusion in the ZNCO electrode at different scanning rates. The ZNCO electrode exhibited ultra-high-rate performance with specific capacities of 259.8 mA h g^−1^ at 1 A g^−1^, and 218 mA h g^−1^ at 50 A g^−1^ and a capacity retention of 83.9%. Qu *et al.* successfully synthesized a novel 3D-layered FeCoNi double hydroxide (FeCoNi-LDH) nanocage with excellent rate performance, grown from a cation exchange reaction with a metal–organic backbone as a template. Its corresponding specific capacity was 980 F g^−1^ at 1 A g^−1^ and the capacity maintains 93% even at a large current density of 20 A g^−1^, indicating that FeCoNi-LDH has promising applications in energy conversion and storage.^113^

### 3.2 Modification

#### 3.2.1 Vacancy defects

Most of the transition metal compounds are challenged by slow electron and ion transport. Therefore, excluding the strategies mentioned before, some other technical means have been investigated to effectively improve the rate performance of electrode materials. It has been proved that vacancy defects can promote electrochemical charge transfer achieving good conductivity and rate performance, because another additional defect level would be generated in the bandgap.^114–116^ A number of fascinating state-of-the-art studies have been reported. For example, Cao *et al.* reported ultra-small oxygen vacancy-rich Co_3_O_4_/graphene composites with ultra-high-rate performance. The specific capacity is able to reach 978.1 F g^−1^ at a current density of 1 A g^−1^ and the capacity retention exceeded 93.7% even at 10 A g^−1^.^50^ Zou *et al.* prepared oxygen vacancy-rich NiMn-LDH based on *in situ* oxidation by an electrodeposition method, reaching a specific capacity of 2150 F g^−1^ at a current density of 1 A g^−1^ and 1518 F g^−1^ at 10 A g^−1^, and a capacity retention of 70.6%, and thus achieving high rate performance.^117^ Feng *et al.* also used electrodeposition to obtain oxygen vacancy-rich NiCo-LDH, which exhibited a specific capacity of 1563 F g^−1^ at a current density of 1 A g^−1^. Particularly, a capacity retention of 67% can be maintained even when applied current density was increased by a factor of 20, suggesting excellent rate performance.^118^

**Fig. 6:**
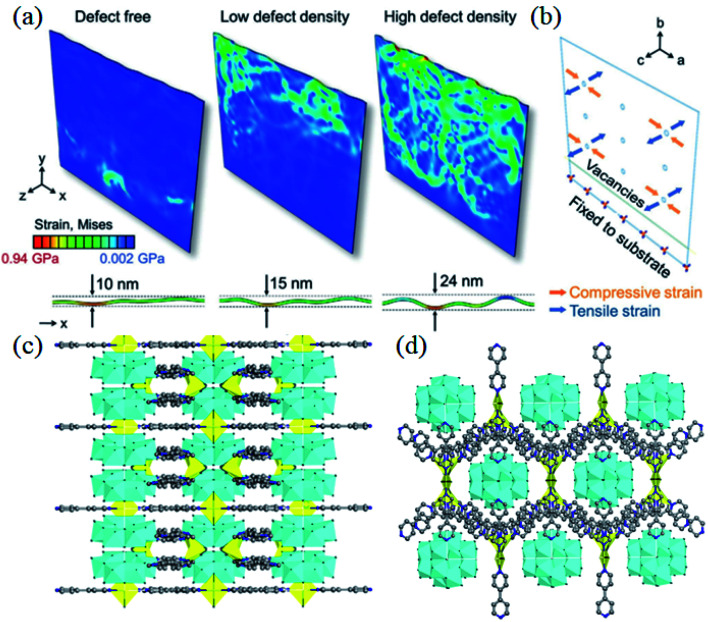
(a) von Mises strain and *c*-axis displacement in nanosheets depicting the corrugation formation process for 0%, 8%, and 18% disordered regions. (b) Schematic of the strain direction on nanosheets during the phase change from α-Ni(OH)_2_ to β-Ni(OH)_2_. Panels a and b adapted with permission from ref. 31, copyright 2020, Wiley-VCH. (c and d) Schematic diagram of the Mo doped Ni-MOF nanosheet structure along the *a*-axis and *c*-axis. Panels c and d adapted with permission from ref. 132, copyright 2020, Elsevier.

Qiu *et al.* activated the intrinsic electrochemical properties of Co(OH)_2_*via* a reverse voltage method that would trigger the phase transition from Co(OH)_2_ to CoOOH. This resulting composition of lattice disorder and continuous cavity molecular structures would greatly facilitate the kinetics. With an abundance of defects, the reactivity of CoOOH would be higher than that of Co(OH)_2_. The electrode material showed excellent rate performance with a specific capacity of 832 F g^−1^ at 1 A g^−1^ and a capacity retention of 78% at 200 A g^−1^.^2^ In addition, they created a special lattice vacancy β-Ni(OH)_2_-based electrode with a unique and distinctive local lattice geometry, as shown in Fig. 6a and b. The tensile strain in the corrugated nanosheets provides an appropriate buffer for the repeated redox reactions, prolonging the lifetime of the electrode at high current densities. The corresponding specific capacity was 746 C g^−1^ at a current density of 1 A g^−1^. Even at 30 A g^−1^, it still had a specific capacity of 457 C g^−1^ with a capacity retention of 61%.^31^Eqn (2) explains the mechanism of this process, where oxygen vacancies and vanadium vacancies generated in the electrodes exhibit superior redox energy barriers, benefitting redox activity and reversible charge storage.

2 4Ni_0.75_V_0.25_(CO_3_^2−^)_0.125_(OH)_2_H_2_O + 2OH^−^ → 3Ni(OH)_2_ + VO_2_ + 6H_2_O + 0.5CO_3_^2−^ + e^−^

#### 3.2.2 Doping

Doping can improve the capacity performance of electrode materials by modulating their conductivity.^119–121^ A lot of research studies report that metal atom doping can increase the conductivity of transition metal oxides by generating additional oxygen vacancies. For example, Chen *et al.* doped Al into cobalt sulfide nanosheets by an electrodeposition method. The appropriate amount of Al doping is able to increase conductivity and enhance the electrochemical activity of cobalt sulfide, resulting in good electrochemical performance. The electrode material had a specific capacity of 2434 F g^−1^ at 1 A g^−1^. It still had 72.3% capacity retention when current density was increased by 100 times.^122^ Ni doped cobalt–cobalt nitride was prepared by Wang *et al.* which showed a specific capacity of 450 F g^−1^ at a current density of 2 mA cm^−2^ and a capacity retention of 82.5% at a current density of 50 mA cm^−2^.^123^ Wei *et al.* doped Mn into Ni–Co layered double hydroxide nanosheets coated by polyaniline-derived carbon. The Mn doping enabled a rich redox reaction (*i.e.* Mn^2+^ ↔ Mn^3+^ ↔ Mn^4+^), which effectively improved the specific capacity and rate performance. A high specific capacity of 1282.06 C g^−1^ and excellent rate stability were observed at a current density of 1 A g^−1^, and the capacity retention was 74.99% when the specific current was increased to 10 A g^−1^.^124^

In addition to the doping of metallic elements into transition metal compounds, the doping of non-metallic elements (O, N, S, F, and P) is also able to increase conductivity.^125–127^ Further, it can enable some other features, including adding the functional groups in the electrodes, improving their hydrophilicity/oil properties, and promoting the transporting efficiency of electrolyte ions.^71,78,79,81,128^ Wang *et al.* doped nitrogen into cobalt phosphide nanowire arrays, which increased the conductivity of cobalt phosphide, improving charge transfer, and giving rise to the high-rate performance of cobalt phosphide. In addition, the doping of N elements increased the number of active sites of the redox reaction in cobalt phosphide, thus increasing the specific capacity. It provides a specific capacity of 232.4 mA h g^−1^ at a current density of 2 mA cm^−2^, and maintains a capacity retention of 52% when the current density is increased to 50 mA cm^−2^.^129^ Meng *et al.* homogeneously doped P into Co–Ni–S nanosheet arrays by an electrodeposition method, which showed an ultra-high specific capacity of 3677 F g^−1^ at 1 A g^−1^ with excellent rate performance (*i.e.* a capacity retention of 63% at 20 A g^−1^).^130^ Wang *et al.* performed sulfur doping by heat treatment and obtained S-doped CoP nanotube arrays with a capacity of 610 F g^−1^ at a current density of 1.0 A g^−1^ and capacity retention of 56% at 20 A g^−1^.^131^ Yang *et al.* encapsulated Mo clusters in the holes of the Ni-MOF frame structure to improve the conductivity and stability of the Ni-MOF (Fig. 6c and d). The nanosheet structure of the Mo-doped Ni-MOF acts as a highway of charge transport, which enables faster charge transportation and the higher conductivity of the electrode material, and thus achieves excellent rate performance. The prepared Mo-doped Ni-MOF electrode exhibits a high specific capacity of 802 C g^−1^ at 1 A g^−1^, and even a specific capacity of 480 C g^−1^ at 10 A g^−1^.^132^

### 3.3 Composite structure

#### 3.3.1 Carbon-based composite structure

Among battery-type electrode materials, NiO is widely used because of its excellent redox and charge storage properties, low cost, and environmental friendliness.^133–135^ However, NiO is also challenged by poor electrical conductivity and cycling stability, which would limit practical applications. To solve this problem, an effective strategy is coupling NiO with other highly porous and conductive materials, such as graphene, activated carbon, and carbon nanotubes because such composite structures can significantly increase the conductivity and improve electrochemical properties. Graphene is a typical 2D material with in-plane carbon atoms arranged in a honeycomb pattern *via* the sp^2^-bond, enabling high electrical conductivity,^136^ a large specific surface area and high theoretical specific capacity.^137^ In addition, its ultrathin 2D structure makes fast charge transfer possible in the plane, whereas its high porosity from the intra- and inter-layer enables fast ion transport.^138,139^ Therefore, the combination of graphene with NiO forming some unique structures is expected to be a desired approach of enhancing the electrochemical performance of electrode materials based on the synergistic effect.^140^ Wang *et al.* synthesized reduced graphene oxide@NiO (rGO@NiO) composites where NiO nanoparticles were uniformly distributed on rGO, and confirmed their ability to strengthen the accessibility from the rGO@NiO composites to the electrolyte.^141^ In addition, the special structure of rGO promotes the fast electron/ion transport.^46,135^ The synergistic effect of rGO and NiO endows the composite electrode with excellent rate performance with a specific capacity of 1093 F g^−1^ at a current density of 1 A g^−1^, and 875 F g^−1^ at 10 A g^−1^ and a capacity retention of 80%.

**Fig. 7:**
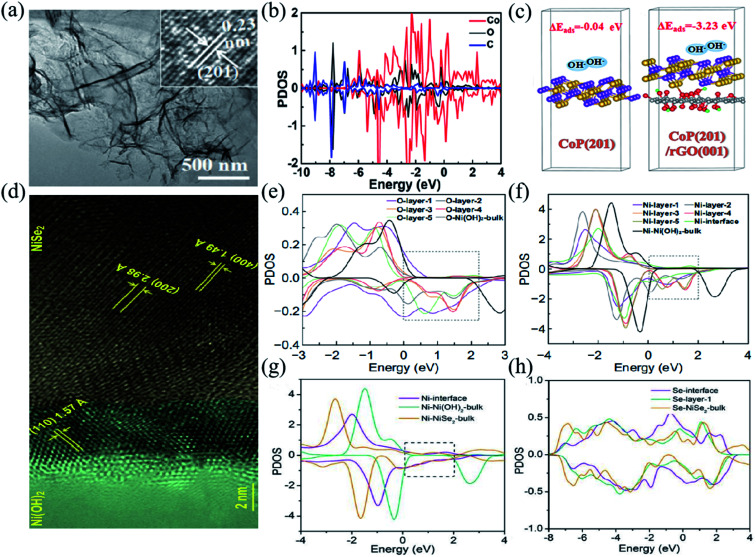
(a) TEM images of the CoP/rGO nanosheets. (b) PDOS of Co, O, and C atoms in CoP (201)/rGO (001). (c) The structure models and the corresponding adsorption energies of OH^−^ for the CoP (201) surface and CoP (201)/rGO (001) interface. Panels a–c adapted with permission from ref. 143, copyright 2021, American Chemical Society. (d) HRTEM images of the NiSe_2_/Ni(OH)_2_ heterojunction. (e and f) PDOS of O and Ni atoms at different layers, respectively. (g) The PDOS of Ni atoms at the Ni(OH)_2_/NiSe_2_ interface, in bulk NiSe_2_, and in bulk Ni(OH)_2_. (h) The PDOS of Se atoms at and near the interface, as well as in the bulk. Panels d–h adapted with permission from ref. 16, copyright 2020, Springer Nature.

Besides NiO, other transition metal compounds can also be coupled with carbon materials achieving high rate performance, such as Ni(OH)_2_/RGO (1795 F g^−1^ at 1 A g^−1^, 85.68% capacity retention at 40 A g^−1^),^142^ PEG/NiCo-DH (2442 F g^−1^ at 1 A g^−1^, a capacity retention of 83.5% at 50 A g^−1^),^49^ NiCo_2_S_4_/PCF (1169 F g^−1^ at 1 A g^−1^, a capacity retention of 81.3% at 20 A g^−1^),^56^ Co_9_S_8_/RGO (1140 F g^−1^ at 4 A g^−1^, a capacity retention of 74.5% at 30 A g^−1^),^19^ and (Ni, Mo)S_2_/RGO (2379 F g^−1^ at 1 A g^−1^, a capacity retention of 60.5% at 100 A g^−1^).^64^ Zheng *et al.* prepared ultrathin, highly crinkled CoP/RGO nanosheet arrays *via* a hydrothermal phosphorylation method (Fig. 7a).^143^ In such CoP/RGO composites, the pores in the nanosheets can serve as active sites for electrolyte diffusion and charge migration, facilitating the redox reaction process, and thus excellent electrochemical properties. Fig. 7b illustrates the partial density of states of the CoP/RGO composite determined by first principles calculations. The strong hybridization between the O atom of RGO and the Co atom of CoP suggests the existence of C–O–Co bonds between the interface of RGO and CoP.^128^Fig. 7c shows the structural model of OH^−^ on the surface CoP (201) and the interface CoP (201)/RGO (001), and the corresponding adsorption energy. The existence of RGO can greatly reduce the adsorption energy of OH^−^ on CoP, thus enhancing the electrochemical performance. The CoP/RGO electrode shows a specific capacity of 1438.0 C g^−1^ at 1 A g^−1^, and 1198.9 C g^−1^ at 10 A g^−1^ and a capacity retention of 83.4%. Moosavifard *et al.* created graphene coated Ni_3_S_2_ nanocube (RGO/Ni_3_S_2_) composites, which can be applied in high-performance and low-cost BSHDs electrodes. RGO/Ni_3_S_2_ electrodes exhibit high rate performance, that is, an ultra-high specific capacity of 616 C g^−1^ at 1 A g^−1^ and a high specific capacity of 377 C g^−1^ at 10 A g^−1^.^144^

#### 3.3.2 Heterojunction composite structure

Building a heterojunction structure based on different electrode materials has been a hot research topic in nano/microstructure engineering, and is able to further optimize the energy storage performance.^145–147^ With built-in fields caused by the mismatch of energy bands from different materials, electron transfer at the interface of the heterojunction would be promoted by an electrostatic force, thus improving the kinetics of redox reactions during charging and discharging.^90,148,149^ Wang *et al.* prepared MgCo_2_O_4_@MnO_2_ core–shell structures by growing MnO_2_ nanostructures on MgCo_2_O_4_ nanoflakes, which showed excellent rate performance (*i.e.* 852.5 F g^−1^ at 1 A g^−1^, and 573.3 F g^−1^ at 40 A g^−1^ and a capacity retention of 67.2%).^53^ Sha *et al.* uniformly deposited Ni(OH)_2_ on silicon carbide nanowires, which promoted its interaction with the electrolyte shortening the ion diffusion distance.^82^ The SiC NWS@Ni(OH)_2_ electrode material showed excellent rate performance, that is, 1724 F g^−1^ at 2 A g^−1^, and 1412 F g^−1^ at 100 A g^−1^ and a capacity retention of 81.9%. Angaiah *et al.* synthesized molybdenum diselenide-nickel hydroxide nanohybrids with molybdenum diselenide grown on nickel hydroxide nanosheets, which was confirmed to have higher conductivity than nickel hydroxide. The rapid electron transfer that occurred at high current densities suggests improved rate performance (*i.e.* 1175 F g^−1^ at 1 A g^−1^, and 1006 F g^−1^ at 10 A g^−1^ and a capacity retention of 85.6%).^70^

Sun *et al.* prepared (100)-NiSe_2_/(110)-Ni(OH)_2_ heterojunction composites using an epitaxial-like growth strategy (Fig. 7d).^16^ Owing to the large specific surface area and suitable microporous structure of the NiSe_2_/Ni(OH)_2_ heterojunction, abundant electrochemically active centers would enable easy access of electrons to the electrolyte and rapid migration of ions inside the electrode. Fig. 7e–h illustrate the electronic density of states of the atoms on the NiSe_2_/Ni(OH)_2_ interface. The NiSe_2_/Ni(OH)_2_ electrode material exhibits excellent electrochemical properties with specific capacities of 909 C g^−1^ at 1 A g^−1^, and 597 C g^−1^ at 20 A g^−1^ and a capacity retention of 63.7%. Zeng *et al.* successfully prepared ZnCo_2_O_4_@MnCo_2_O_4_ heterojunction structure nanosheets with several advantages, including a large specific surface area, good structural stability and good electrical conductivity, exhibiting high-rate performance. The corresponding specific capacity is 254 F g^−1^ at 1 A g^−1^, and it can remains 186 F g^−1^ even at 10 A g^−1^.^150^ Li *et al.* fabricated the α-Ni(OH)_2_/NiS_1.97_ heterojunction by an ion-exchange based epitaxial growth method. Heterojunctions allow the components to provide ion/electron transport paths for each other, increasing the utilization rate of active materials, and thus achieving high specific capacity and rate performance. The corresponding specific capacity is as high as 2375.8 F g^−1^ at 1 mV s^−1^ and 939.5 F g^−1^ at 10 mV s^−1^.^151^

### 3.4 Array structure

**Fig. 8:**
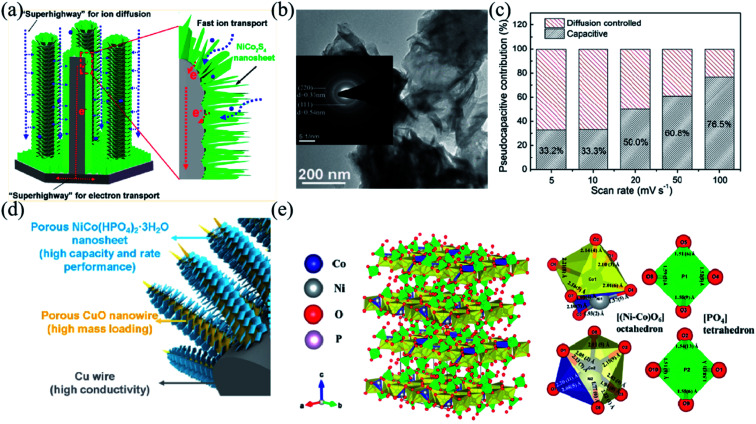
(a) Schematic illustration of ion diffusion and electron transport in the vertically aligned NiCo_2_S_4_@NC-array. (b) Transmission electron microscopy image of NiCo_2_S_4_ Nanoflakes. (c) Ratio of pseudocapacitive contribution at different scan rates. Panels a–c adapted with permission from ref. 161, copyright 2020, American Chemical Society. (d) Structure advantages of the Cu/p-CuO/NiCo-P hybrid material. (e) 3D atomic configuration of a NiCo(HPO_4_)_2_–3H_2_O unit cell and the local coordination of the strongly distorted [(Ni–Co)O_6_] octahedron and [PO_4_] tetrahedron. Panels d and e adapted with permission from ref. 163, copyright 2020, American Chemical Society.

Since the quality of the electron transport channel is crucial to the performance of electrode materials, different morphologies of nanomaterials usually alter electron transporting properties by directly affecting the specific surface area and density of electrode materials.^55,91,152,153^ Therefore, a reasonable design of the electrode structure plays an important role in the development of advanced electrode materials for high-performance BSHDs.^99,129,154^ 3D array structures are found to increase the specific surface area and density of electrode materials, which would provide abundant electrochemical activity centers, facilitating charge transport, and consequently strengthening their electrochemical performance.^54,154–157^ Therefore 3D array structures are considered attractive for maximizing the use of active materials.^158–160^ Wang *et al.* prepared high-performance electrode materials by depositing arrays of copper hydroxide nanosheets on nickel grids *via* electrodeposition.^157^ They have a specific capacity of 798.2 F g^−1^ at 1 A g^−1^ and a specific capacity of 556.4 F g^−1^ at 20 A g^−1^ with a capacity retention of 70%. As copper hydroxide nanosheets have a large specific surface area which can sufficiently contact the electrolyte during the charging and discharging process, the length of the ion diffusion path would be shortened and the “dead volume” in the electrode would greatly reduce. In addition, the *in situ* growth method avoids the use of polymer binders, which significantly improves electron transfer and ion diffusion.^48,68,103^ Chen *et al.* reported a composite electrode consisting of a vertical, ordered arrangement of NiCo_2_S_4_ nanoflakes covered by elongated nickel columns (Fig. 8a).^161^ The array structure enabled fast charge storage/transfer and promoted reaction kinetics, allowing the active materials to react efficiently. The transmission electron microscopy (TEM) image in Fig. 8b shows intersecting ultrathin NiCo_2_S_4_ sheets forming a sparse nanostructure. The contribution rate of the NiCo_2_S_4_@NC array electrode at different scan rates is shown in Fig. 8c. With the increment of the scan rate, the contribution from diffusion decreases. The shorter ion diffusion time at high scan rates results in inadequate redox reactions. In this case, the capacitive control occurring on the material surface would dominate. Due to the good charge transfer kinetics and fast electron transfer, the NiCo_2_S_4_@NC array electrode has an ultra-high specific capacity of 486.9 mA h g^−1^ at 1 A g^−1^ and 150 mA h g^−1^ even at a very high current density of 100 A g^−1^.

Transition metal phosphides have excellent electrical conductivity and redox activity due to the lower electronegativity of phosphorus, as mentioned before.^42,44,45,162^ Chen *et al. in situ* grew dense NiCo(HPO_4_)_2_–3H_2_O nanosheet arrays on porous CuO nanowires, referred to as Cu/p-CuO/NiCo-P, and the corresponding schematic diagram is shown in Fig. 8d.^163^Fig. 8e suggests that the single layer of NiCo(HPO_4_)_2_–3H_2_O consists of octahedra [(Ni–Co)O_6_] and tetrahedra [PO_4_]. Cu/p-CuO nanowires would increase the conductivity of the whole electrode materials as the conducting frame and provide a large specific surface area for high-quality loaded active substances. The synergistic effect of Ni and Co effectively improves the electrical conductivity and capacitive properties of NiCo-P. The abundant pores and defects of ultrathin NiCo-P nanosheets can effectively expose the active centers promoting ion diffusion and charge transfer. The Cu/p-CuO/NiCo-P electrode exhibits ultra-high specific capacity (*i.e.* 1768.5 C g^−1^ at 1 A g^−1^) and excellent rate performance (*i.e.* 1144.8 C g^−1^ at 100 A g^−1^, 64.7% capacity retention).

### 3.5 Interface engineering

Since the interface of electrode materials is an important region where electrochemical reactions occur, the capacitive performance of BSHD electrode materials strongly depends on effective electron and ion transport at the interface. Therefore, building effective interfaces inside composite electrodes or between the electrode/electrolyte is key to improving the electrochemical energy storage performance.^92^ Interfaces between a metalloid/semiconductor would form Schottky contact, which can facilitate electron transfer from the free-electron-rich metalloid to the semiconductor and leave holes in the metalloid, thus enhancing OH^−^ adsorption.^164^ In addition, the interface will release additional electrons during charging and discharging, ultimately improving the electrochemical performance of the electrode.^165,166^ Such interface engineering has been verified as an effective approach of improving the conductivity and structural stability of electrodes in SBHs by many previous studies.^167–169^ For example, Du *et al.* reported a NiCo_2_O_4_ nanowires/reduced graphene oxide (RGO) electrode material, which is able to combine the energy storage mechanisms of capacitive and Faraday materials.^165^ With the interconnected porous skeleton of the RGO/NiCo_2_O_4_ nanocomposite and the polarization of the interface caused by the difference of work function, the kinetics of the electronic and ionic reactions are enhanced, the structural stability is improved, and the rate performance of the RGO/NiCo_2_O_4_ nanostructure is greatly enhanced, that is, 4.37 F cm^−2^ at 2 mA cm^−2^ and 2.59 F cm^−2^ at 10 mA cm^−2^ with 67.5% capacity retention. Gao *et al.* proposed a simple interface engineering strategy where 2D MoS_2_ nanosheets were *in situ* integrated into a 3D polypyrrole framework *via* a DBS-anion intermediate linkage, consequently forming a novel MoS_2_-DBS-PPy film.^168^ As such the MoS_2_-DBS-PPy film combined the excellent conductivity of PPy chains and the high specific surface area of MoS_2_ nanosheets, and exhibited excellent rate performance (*i.e.* 1.2 F cm^−2^ at 0.5 mA cm^−2^, and 0.6 F cm^−2^ at 10 mA cm^−2^ with 50% capacity retention).

**Fig. 9:**
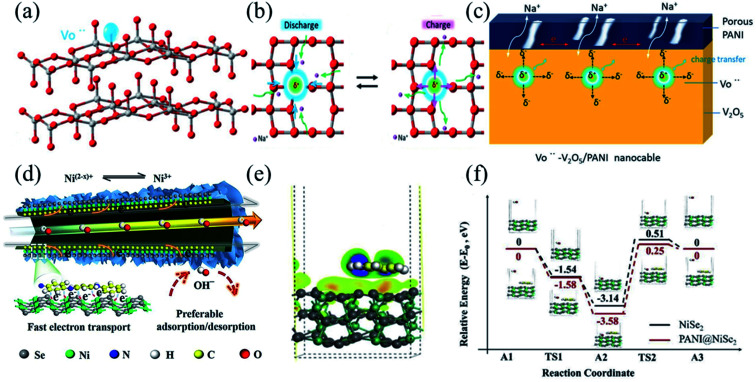
(a) The vacancy site in the V_2_O_5_ structure. (b) Schematic illustration of charge transfer behavior around the Vö region by forming a local electric field (*E*). (c) The enhanced charge transfer kinetics mechanism of Vö–V_2_O_5_/PANI due to the synergy of Vö and PANI. Panels a–c adapted with permission from ref. 175, copyright 2019, WILEY-VCH. (d) The electron transport mechanism of PANI@NiSe_2_. (e) The electronic density difference of PANI@NiSe_2_. (f) The DFT calculations of the transition-state energy of adsorbed hydroxyl (Δ*G*_OH^−^_). Panels d–f adapted with permission from ref. 176, copyright 2019, Elsevier.

With the capability of strengthening the conductivity and stability of electrode materials by forming a highly conductive layer,^170,171^ polyaniline is widely implemented in energy devices by coupling with pseudocapacitive electrode materials.^172–174^ Cao *et al.* synthesized V_2_O_5_/PANI nanomaterials *via in situ* one-step oxidative polymerization of aniline monomers, which will insert vanadium vacancies and PANI on the surface of V_2_O_5_ (Fig. 9a).^175^ With the tilting of vanadium atoms, an unbalanced charge distribution generates in the plane, thus forming a local electric field which would provide the Coulomb force facilitating ion diffusion (Fig. 9b). Vanadium vacancies enable fast electron transfer by providing vacancy sites for electron adsorption/desorption, and also catalyze redox reactions on the surface. In addition, as an external percolation charge-transporting channel in the nanostructure, the high conductivity of the porous polyphenylene shell layer accelerates electron transport (Fig. 9c). Therefore, with vanadium vacancies and the interface modification of PANI, the charge transfer kinetics of the V_2_O_5_/PANI electrode would be enhanced, resulting in excellent electrochemical performance. It exhibited a specific capacity of 353.8 F g^−1^ at 1 A g^−1^ and a capacity retention of 50% at 10 A g^−1^. Yang *et al.* fabricated a PANI/SeNi_2_ core–shell nanotube electrode with different electron concentrations (Fig. 9d).^176^ The strong interaction between PANI and SeNi_2_ through the CNI–Se bond can induce electron accumulation at the Ni ion, which not only promotes electron transfer within the electrode, but also increases the theoretical Faraday capacity of the electrode. The Faraday redox reaction occurring at the electrode follows eqn (3):

3 PANI@NiSe_2_ + (1 + *x*)OH^−^ ↔ NiSe_2_(OH)_1+*x*_ + (1 + *x*)e^−^

Fig. 9e illustrates the transition state energy of the adsorbed hydroxyl group (Δ*G*_OH^−^_) calculated by DFT. Compared to NiSe_2_, the strong interaction through the CNI–Se bridge bond at the interface of PANI/NiSe_2_ promoted the electron transfer and the adsorption/desorption of ions on the surface, as shown in Fig. 9f. The combination of NiSe_2_ and PANI induced electron accumulation from lower valence states or a wide range of valence changes of Ni ions, which improved the specific capacity and rate performance of the electrode material. The electrode had a specific capacity of 275.6 mA h g^−1^ at 1 A g^−1^ and a capacity retention of 61% at 10 A g^−1^.

### 3.6 Catalytic effect

As catalyst materials play a crucial role in redox reactions during electrochemical energy conversion and storage,^177–179^ their activity and stability would determine the electrochemical performance of energy storage devices. Thus, it makes sense to study the catalytic effect of electrode materials, further enhancing the rate performance and cycling stability of electrode materials.^180,181^ Transition metal sulfides have received great attention in the application of energy conversion and storage devices due to their high specific capacity and rate performance.^182–184^ Guo *et al.* prepared 3D Ni_9_S_8_/O–MoS_2_ nanostructures used in supercapacitors and hydrogen precipitation catalysts. Ni_9_S_8_ nanorods acted as conducting channels of electrons in the Ni_9_S_8_/O–MoS_2_ nanocomposites, enhancing electron transport throughout the electrodes, and thus achieved high electrochemical performance. It exhibited a specific capacity of 907 F g^−1^ at 2 A g^−1^ and retained a specific capacity of 430 F g^−1^ at 7 A g^−1^, enabling good rate performance. In addition, the oxygen doping of MoS_2_ would offer more active sites, which would participate in the catalytic process of the hydrogen precipitation reaction. Therefore, the Ni_9_S_8_/O–MoS_2_ composites also had highly electrocatalytic properties, and can be applied for hydrogen production.^185^

**Fig. 10:**
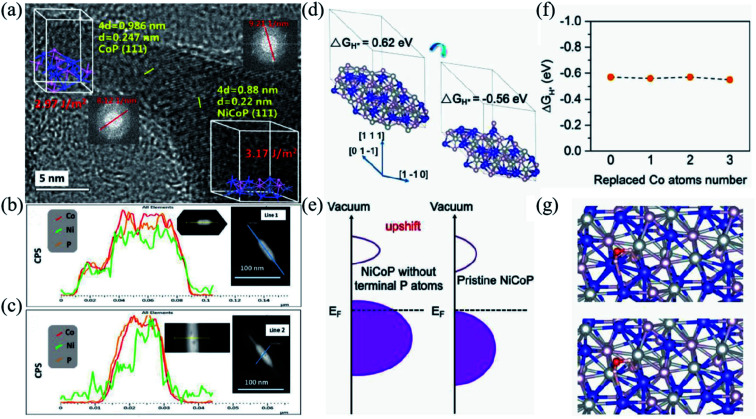
(a) HRTEM image of CoP and NiCoP; the inset shows the calculated surface energies and fast Fourier transform. (b and c) Line scanning in different directions of CoP/NiCoP. (d) The optimized hydrogen atom adsorption structure. (e) The schematic on the projected density of states. (f) The Δ*G*_H^+^_ values as the replaced Ni atoms increased. (g) The optimized hydrogen H_2_O adsorption structure. Panels a–g adapted with permission from ref. 192, copyright 2019, WILEY-VCH.

Transition metal phosphides are considered a kind of stable and effective catalyst material because of their unique catalytic and electronic properties. Owing to the electron-rich metallic surfaces of transition metal phosphides, their activity is better than that of other counterparts, such as carbides, nitrides, and sulfides.^186–188^ Thus, transition metal phosphides are widely used in water decomposition, fuel cells, batteries, and supercapacitors.^189,190^ Pang *et al.* prepared ultrathin and homogeneous 2D nickel–cobalt phosphate composite nanosheets, which are ultrathin enough to provide maximum mechanical flexibility and sufficient electroactive sites facilitating their electrochemical performance. The electrode shows a high specific capacity of 1132.5 F g^−1^ at 1 A g^−1^ and high rate performance with 63% capacity retention at 1 A g^−1^.^191^ Chen *et al.* built a nanoscale tadpole-like CoP/NiCoP heterojunction interface modulating the catalyst structure and thus strengthening the hydrogen precipitation and energy storage performance, as shown in Fig. 10a. Fig. 10b and c demonstrate linear sweeping in different directions, further revealing the elementary distribution of CoP/NiCoP. Density functional theory (DFT) explains the synergistic effect between NiCoP and CoP, confirming the effective proton adsorption/desorption ability of CoP/NiCoP (Fig. 10d). The d-band center of the H^+^ capture site would shift upward from the Fermi energy level after the elimination of the terminal P atoms (Fig. 10e). After CoP loading, the coordination number of the Co–Ni shell layer in NiCoP would decrease, and some of the Ni atoms would be replaced by Co atoms on the surface. The magnitude of Δ*G*_H^+^_ remains almost constant with the increased substitution of Ni atoms (Fig. 10f). In addition, the adsorption energy of H_2_O (Δ*E*_H_2_O_) was calculated and is illustrated in Fig. 10g, which indicates that in the alkaline HER process the adsorption of H_2_O would be improved and the dissociation is accelerated. The theoretical results show that the formation of the NiCoP/CoP heterojunction interface optimizes proton chemisorption and H_2_O dissociation. The optimized phosphide crystal keeps the balance of the proton chemisorption and H_2_O dissociation, thus accelerating the HER kinetics over a broad pH range. In addition, because of the unique properties, CoP/NiCoP had been proved as an excellent cathode material for SBHs with ultra-high rate performance, which was a specific capacity of 1106.2 F g^−1^ at 1 A g^−1^ and a specific capacity of 600 F g^−1^ at 100 A g^−1^.^192^

## 4. Conclusion and outlook

As a new generation of energy storage devices, BSHDs with high energy density, power density, and cycling stability have a wide application prospect. Due to the poor conductivity and structural stability, and the slow ion diffusion kinetics, the rate performance of transition metal compound-based cathodes cannot meet the demand, limiting the practical application of BSHDs. To solve this problem, this review introduces the energy storage mechanism of BSHDs and summarizes some efficient strategies of reinforcing the rate performance of transition metal compound-based cathodes. It may put forward a reference for developing new strategies to further improve the rate performance of transition metal compound-based cathodes in BSHDs.

With respect to the future design and fabrication of BSHDs, a number of points are still worth considering; the corresponding outlooks are listed below:

### 4.1 Optimizing the experimental method

In the preparation of transition metal sulfide/selenide/phosphate, toxic gases such as H_2_S, H_2_Se and PH_3_ may be produced, which are hazardous to human health. In addition, phosphorus-containing waste solution produced from the synthesis process needs to be carefully handled; otherwise it would cause environmental pollution. Therefore, the optimization of the preparation method is necessary.

### 4.2 Developing biomass-derived carbon materials

The complex synthesis process and high cost of graphene limit the wide application of transition metal compounds and graphene composites. Therefore, developing transition metal compound coupled biomass-derived carbon materials could be an effective strategy.

### 4.3 Amorphizing the surface

Amorphization is a process of making order crystals structurally amorphous. For electrode materials in SBHs, surface amorphization modulates the physicochemical properties, resulting in better electrochemical performance. Currently, most electrode materials are based on crystalline or layered compounds with good orderly crystallinity. However, a disorder part of the surface will be able to enhance the specific capacity of the material, and greatly increase the diffusion efficiency of ions, thus greatly improving the rate performance.^193^

### 4.4 Optimizing the electrolyte

Electrolyte is an important part of supercapacitors, whose interaction with the electrode would determine the electrode–electrolyte interface state, further having an impact on the rate performance. For example, aqueous electrolytes have high ionic conductivity, but are limited by low energy density and poor cycling stability. Organic electrolytes and ionic liquids usually have low ionic conductivity, although they can expand the potential window of electrode materials. Although water-in-salt electrolytes have a wide electrochemical window, making them promising electrolytes, their low conductivity and high viscosity limit their further application in SBHs. Therefore, there is a need for the design and development of more efficient electrolytes for SBHs.^194^

## Author contributions

Cong Wang and Zehao Song wrote this manuscript; Pei Shi, Lin Lv, Houzhao Wan, Li Tao, Jun Zhang, Hanbin Wang, and Hao Wang supervised the work and revised the manuscript.

## Conflicts of interest

We declare that we do not have any commercial or associative interest that represents a conflict of interest in connection with the work submitted.

## Supplementary Material
